# Scene construction deficits in adolescent PTSD are in sensory, rather than spatial, imagery

**DOI:** 10.3389/fpsyg.2025.1589756

**Published:** 2025-08-06

**Authors:** Hannah Marlatte, Jennifer D. Ryan, Asaf Gilboa

**Affiliations:** ^1^Department of Psychology, University of Toronto St. George, Toronto, ON, Canada; ^2^Rotman Research Institute, Baycrest Health Sciences, Toronto, ON, Canada; ^3^Department of Psychiatry, University of Toronto St. George, Toronto, ON, Canada

**Keywords:** post-traumatic stress disorder, adolescence, scene construction, mental simulation, hippocampus

## Abstract

**Introduction:**

Post-traumatic stress disorder (PTSD) is characterized by memory and imagery disturbances, ascribed in part to structural and functional hippocampal abnormalities. These include impaired mental simulation of past and future events, as well as deficits in imagining novel, neutral, spatially coherent scenes (“scene construction”). Structural hippocampal differences are less consistently found in adolescent PTSD; however, deficits in recalling specific autobiographical memories have been noted.

**Methods:**

We examined scene construction ability in adolescents with PTSD, a presumably hippocampal-dependent process. Forty adolescents were recruited through the community: 26 with diverse trauma exposure (7 with PTSD, 19 without PTSD), and 14 non-trauma-exposed healthy controls. Scene construction performance was compared across groups using non-parametric ANOVAs and was related to PTSD symptom severity regardless of group membership using linear regressions.

**Results:**

No differences in global scene construction performance were found; however, adolescents with PTSD imagined a smaller proportion of sensory details than control groups. Cognitive ability predicted several aspects of scene construction performance, rather than PTSD severity, as had been expected based on the adult literature. Nonetheless, those higher in avoidance symptoms imagined more person-related details, and trauma-exposed participants also reported feeling more present within their imagined scenes compared to healthy non-trauma-exposed controls.

**Discussion:**

Together, these results suggest that hippocampal-dependent deficits in scene construction as seen in adults are not apparent in adolescence, however, changes in sensory imagery are. These findings provide broader insights into PTSD-related cognitive changes during development and inform interventions for this population that focus on sensory experiencing to promote embodiment, even within one’s “mind’s eye”.

## 1 Introduction

Adolescence has been identified as a period during which youth are at a greater risk for exposure to trauma and, therefore, at greater risk for developing PTSD (Kilpatrick et al., 2003). In fact, most adolescents experience a traumatic event by the age of sixteen (Copeland et al., 2007; McLaughlin et al., 2013). Adolescent PTSD is associated with memory disturbances, such as the hallmark symptom of intrusive re-experiencing of past traumatic events. However, the research on the umbrella cognitive function of mental simulation—defined as the ability to bring to mind alternate temporal, spatial, or hypothetical realities—is limited and inconsistent. The goal of the current study was to examine one form of mental simulation in adolescent PTSD called scene construction (Hassabis and Maguire, 2007). This process involves mentally constructing novel, neutral scenes and has recently been found to be impaired in adult-onset PTSD, which was associated with having smaller hippocampal volumes (Marlatte et al., 2022). As reviewed below, while hippocampal structural changes associated with PTSD may not be evident in adolescence, behavioral deficits in hippocampal-dependent processing are still observed. Thus, a behavioral examination of scene construction performance in adolescent PTSD is warranted.

To date, most studies on mental simulation ability in adolescents with PTSD have focused on impairments in future thinking. Future thinking impairments may be a critical factor in the development of PTSD as deficits are associated with cognitive and behavioral changes that can lead to either trauma exposure or the later development of psychopathology. Conducting more high-risk behavior is common in adolescence (Nooner et al., 2012) and may be related to experiencing a greater number of traumatic events (Layne et al., 2014). Risky behavior is also associated with (i) spending less time thinking about the future, including setting goals and plans (having a “future orientation”; Johnson et al., 2014), as well as (ii) impairments in vividly imagining future personal events (“episodic future thinking”; Bromberg et al., 2015). Adolescents who are less future-oriented also show greater feelings of hopelessness, lower overall well-being, and a higher likelihood to utilize maladaptive coping skills (Chua et al., 2015; Mac Giollabhui et al., 2018) which can increase the likelihood of developing psychopathology after trauma exposure. Impairments in mental simulation more broadly, including both past and future personal events, are also present in adulthood if PTSD is acquired (Brown et al., 2013, 2014; Sutherland and Bryant, 2007) such that simulated events may be lacking specific contextual information and therefore be “over-general”. Children and adolescents exposed to trauma also report over-generalized autobiographical memories (De Decker et al., 2003; Nixon et al., 2013; Crane et al., 2014), although findings are at times inconsistent (for review, see Hitchcock et al., 2014). Despite the potential relationship between the development of trauma-related psychopathology and deficits in mental simulation, including future episodic thinking, to our knowledge, it has not yet been examined directly in adolescents with PTSD.

Although there is limited and mixed behavioral research on mental simulation in adolescent PTSD, mental simulation ability involves neural structures commonly associated with the disorder, such as fronto-limbic circuits that include the hippocampus and ventral medial prefrontal cortex (vmPFC; Ciaramelli et al., 2021; McCormick et al., 2018). Although perspectives differ on exactly how the hippocampus supports mental simulation, there is broad agreement that it is critical for constructing mental representations composed of multiple elements (Hassabis and Maguire, 2007; Olsen et al., 2012; Schacter and Addis, 2007) through connections with the vmPFC (Campbell et al., 2018; McCormick et al., 2020; Monk et al., 2020). One paradigm specifically assesses the ability to construct multimodal scene imagery, defined as naturalistic and spatially coherent representations typically populated with objects (“scene construction”; Hassabis et al., 2007). Unlike other kinds of mental simulation, such as past or future episodic thinking, which are more explicitly tied to specific time orientations, scene construction may be less temporally constrained (i.e., involving imagining both personal future and fictitious scenarios). Nevertheless, patients with lesions to either the hippocampus (Hassabis et al., 2007) or vmPFC (Bertossi et al., 2016) have been shown to be profoundly impaired at imagining such detail-rich and spatially coherent scenes. Adults with PTSD have been found to show similar impairments, with more severe scene construction deficits being associated with smaller hippocampal volumes (Marlatte et al., 2022). This aligns with previous literature on adult PTSD and mental simulation more broadly, in which reduced hippocampal volumes are consistently noted (Karl et al., 2006; Kitayama et al., 2005; Logue et al., 2018; Woon et al., 2010) and are thought to contribute to the frequently reported deficits in episodic memory and future thinking (Brown et al., 2013, 2014; Kleim et al., 2014; Kleim and Ehlers, 2008; Ono et al., 2016; Sutherland and Bryant, 2007).

Although reduced hippocampal volumes are commonly reported in adults with PTSD, evidence in childhood and adolescence are mixed: some studies note smaller overall hippocampal (Carrión et al., 2007) and subregion volumes (Postel et al., 2019) in children and adolescents with PTSD, whereas other studies have noted no differences (Ahmed et al., 2012; Carrión et al., 2001; De Bellis et al., 2002; Morey et al., 2016) or even larger volumes in those with PTSD (Tupler and De Bellis, 2006). A meta-analysis of hippocampal volumes in both children and adults who experienced childhood maltreatment suggests PTSD-related hippocampal volume differences observed in adulthood may not yet be apparent in adolescence (Woon and Hedges, 2008), perhaps due to delayed pathological expression. Nonetheless, PTSD symptoms in adolescence are predictive of later hippocampal volume loss (Carrión et al., 2007) and functional differences are also present: adolescents with PTSD show altered hippocampal-default mode network connectivity that improves with symptom reduction (Sussman et al., 2022) and trauma-exposed children show less hippocampal activation during a memory task (Carrión et al., 2010). Neuropsychological deficits, including pervasive executive dysfunction, impaired learning and problem solving, and susceptibility to distraction and impulsivity, in pediatric PTSD parallel with those seen in adults (Beers et al., 2002; Moradi et al., 1999; Saigh et al., 2006; Samuelson et al., 2010), which may be due to dysfunction in frontal regions rather than the hippocampus. Indeed, adolescent PTSD is associated with abnormal frontolimbic development, including having smaller vmPFC volumes (Morey et al., 2016; Carrión et al., 2001).

Together, the current literature suggests that while PTSD-related structural changes in the hippocampus may not yet be evident in adolescence, adolescents with PTSD tend to show behavioral impairments in cognitive processes that are hippocampal-dependent, as well as in executive functioning due to structural changes in prefrontal regions. The goal of the current study was to examine the ability of adolescents with PTSD to imagine spatially coherent neutral scenes—a presumably hippocampal-dependent cognitive process that also implicates the vmPFC—using a scene construction task (Hassabis et al., 2007). We expected to find similar scene construction deficits in adolescent-onset PTSD as has been previously seen in adults (Marlatte et al., 2022). Specifically, we predicted greater PTSD severity would be associated with impairments in scene construction ability as indexed by the number and kinds of details imagined, the spatial coherence among details, and the quality of imagined scenes as rated by an external scorer and through participant’s subjective ratings of salience and presence. Findings from this study will provide broader insights into PTSD-related cognitive changes during development and inform interventions of particular relevance for this population.

## 2 Materials and methods

### 2.1 Participants

Forty adolescents between the ages of 11–18 participated in the study (Table 1). Participants were recruited from the community in the Greater Toronto Area through online and flyer advertisements: some advertisements were targeted towards the recruitment of neurologically and psychiatrically healthy individuals whereas others were targeted towards the recruitment of those experiencing traumatic memories. All participants provided written consent in compliance with the Baycrest Research Ethics Board, received cash compensation, and underwent an initial screening to ensure eligibility.

**Table 1:** Participant demographics and clinical summary.

Variable	PTSD	Trauma-exposed controls	Healthy controls	Sig.
*N* (female)	7 (6)	19 (13)	14 (11)	
Age	16.29 (1.50)	16.05 (1.75)	14.79 (1.48)	
Education	10.43 (2.15)	10.21 (1.93)	9.00 (1.52)	
CPSS-V	49.86 (12.02)	10.79 (10.63)	2.36 (4.70)	***
BDI-II	24.71 (8.92)	11.84 (10.72)	11.93 (8.91)	*
STAI-state	47.71 (9.11)	38.32 (11.62)	37.07 (10.97)	
STAI-trait	58.14 (8.13)	44.74 (11.00)	41.57 (9.99)	**
BSI-53 – global severity index	2.15 (0.60)	0.80 (0.60)	0.66 (0.46)	**
Shipley – vocabulary	27.29 (6.95)	26.05 (5.21)	27.64 (3.90)	
Shipley – abstraction	28.28 (9.70)	29.58 (8.56)	30.14 (8.06)	

*p < 0.05, **p < 0.01, ***p < 0.001.

Inclusion criteria for all participants were being 11–18 years old and fluent in English. Participants in the trauma-exposed and PTSD groups were to also have experienced a Criterion A traumatic event, and could have comorbid anxiety disorders, obsessive-compulsive disorder, and/or depression. Exclusion criteria for all participants were having experienced a traumatic brain injury where consciousness was lost for more than 5 min, recently initiated (within 3 months) or adjusted (within 6 weeks) treatment with psychotropic medications, and prior or current experience of psychosis or a substance-use disorder. Healthy controls were to also have no history of psychiatric diagnosis or taking psychotropic medication. Participants were designated into a trauma-exposed group if they had experienced a Criterion A traumatic stressor. Such events included experiencing or witnessing physical assault (*n* = 18), experiencing sexual assault (*n* = 7) or a disaster or accident (*n* = 5), sudden or threatened death to someone close (*n* = 6), a violent act such as a school shooting (*n* = 6), or a stressful medical procedure (*n* = 3). Most of these participants had experienced more than one event (see Supplementary Materials for a summary of CPSS-V subscales).

An *a priori* power analysis was conducted using G*Power version 3.1 (Faul et al., 2009) for sample size estimation based on our previous study of adults with PTSD who were robustly impaired at this task (Cohens *d* = 2.15; Marlatte et al., 2022). To find the same effect using an ANOVA test, 6 adolescents per group would provide 95% power to detect scene construction performance differences at a 5% alpha-level. Additionally, 24 total participants would provide 95% power to detect a relationship between scene construction performance and trauma symptom severity through linear multiple regression. A more conservative power estimate of 0.95 was chosen due to the large effect sizes seen in our previous study and oversampling was done to achieve both criteria.

### 2.2 Stimuli and procedure

After providing informed consent, participants first completed the scene construction task (Hassabis and Maguire, 2007). Here participants were instructed to vividly imagine and then describe a series of commonplace scenes, consisting of seven fictitious scenarios and two personal future events (e.g., “Imagine you are lying on a white sandy beach in a beautiful tropical bay. I want you to describe the experience and the surroundings in as much detail as possible using all your senses, including what you can see, hear, and feel”). One prompt from the original paradigm was not included as it was a narrative, and another was adapted to be suitable for this age group. For each scene, participants were explicitly asked not to recall an actual memory but to create something new. They were also instructed to continue with their descriptions until they came to a natural end or felt like nothing else could be added. After describing each scene in as much multimodal detail as possible, participants rated the scene on their perceived scene of presence, salience, and spatial coherence on a computer through a survey using Qualtrics software (Qualtrics, 2018). Each participant was tested individually and faced the interviewer, who read aloud the instructions for each scenario from a prepared script and provided prompts to aid in detail generation when needed. Participants’ narratives were recorded and later transcribed for scoring, and a practice trial was completed beforehand to ensure understanding of the task (see Supplementary Materials for all prompts, subjective ratings, and a scoring sample).

After completing the scene construction task, participants completed a series of self-report symptom assessments and measures of crystalized and fluid intelligence on a computer using Qualtrics software (Qualtrics, 2018). Participants first completed an assessment for PTSD symptom severity using the Child PTSD Symptom Scale for DSM-V (CPSS-5) with Trauma Screening (Foa et al., 2018). This provides a total score of symptom severity, comprised of a sum of four subscales that align with the DSM criterion, as well as a measure of the severity of impairment in everyday functioning, which does not contribute to the overall score. Non-trauma-exposed controls reported symptoms based on their most stressful experience that did not qualify as a Criterion A stressor. Afterwards, participants completed the Beck Depression Inventory (BDI-II; Beck et al., 1996), the State–Trait Anxiety Inventory (STAI; Spielberger, 2010), and the Brief Symptom Inventory (BSI-53; Derogatis and Melisaratos, 1983), the latter of which provides a measure of overall psychological distress through the Global Severity Index. Finally, participants completed tests of vocabulary and abstraction ability through the Shipley’s Institute of Living Scale (Shipley, 1940).

Scene construction narratives were transcribed from recordings and coded by an external scorer to quantify each detail type, total detail count, and to provide an overall quality rating. The first author coded a subset (20%) to assess inter-rater reliability, with good to excellent reliability found for all items (ICC = 0.85–0.96). To measure the overall richness of each imagined scene, a composite score called the Experiential Index was also calculated using normalized scores of objective scene content, subjective ratings of scene quality and spatial coherence by the participant, and quality judgements by the scorer. For one participant, the BSI-53 could not be collected and is therefore missing.

### 2.3 Analyses

Two analytical approaches were taken: the first was to assess group-wise differences in scene construction performance, similar to our previous study (Marlatte et al., 2022). This was done through a series of non-parametric one-way ANOVAs as the group sizes were unequal. Given the dimensional nature of psychopathology, we also examined the relationship between symptom severity and scene construction performance for all participants regardless of group designation through a series of multiple linear regressions. All analyses were completing using R (version 4.4.1; R Core Team, 2024) and R Studio (version 2024.02.2; Posit Team, 2024).

Kruskal-Wallis tests were run to compare group-wise differences, using eta-squared as a measure of effect size. When needed, post-hoc analyses were completed using Dunn’s Multiple Comparison Test with adjusted *p*-values, with effect size measured using Pearson’s r. Such comparisons were completed for all symptom assessments and measures of cognitive ability, as well as performance in the scene construction task. Omnibus tests were calculated using the stats package (version 4.2.1; R Core Team, 2024), and omnibus effect sizes and post-hoc comparisons calculated using the rstatix package (version 0.7.2; Kassambara, 2023).

To examine the relationship between dimensions of PTSD symptom severity and task performance, regressions were run using the stats package (version 4.2.1; R Core Team, 2024) predicting all aspects of scene construction performance: overall performance through the Experiential Index, as well as individual measures of mean presence, salience, and spatial coherence of the scenes, the average number of total details and each detail type, and overall scene quality. Predictors and covariates were selected based on their theoretical relevance to mental simulation. Predictors consisted of the subscales of the CPSS-V assessing different aspects of PTSD symptomatology: arousal and reactivity, avoidance, changes in cognition and mood, and experience of intrusions. Covariates included depression symptom severity (BDI-II), overall distress related to their psychopathological symptoms (The Global Severity Index from the BSI-53), and overall cognitive ability (Shipley total score).

## 3 Results

### 3.1 Demographics and psychopathology

Groups did not differ based on age, education, or gender. By definition, adolescents with PTSD reported greater PTSD severity than control groups, and trauma-exposed controls reported greater symptom severity than healthy controls. The PTSD group also had higher depression symptoms, trait anxiety, and overall distress related to their psychopathological symptoms than the control groups, which did not differ from each other. No differences between groups were noted for state anxiety or aspects of cognitive ability. See Supplementary Material for full statistical reporting.

### 3.2 Group-wise comparisons

See Table 2 for a summary of results. No group differences were found for overall scene construction performance through the Experiential Index, χ^2^(2) = 1.79, *p* = 0.401, for total details within the narratives, χ^2^(2) = 1.66, *p* = 0.436, or for overall quality as rated by an external scorer, χ^2^(2) = 2.80, *p* = 0.246. For specific detail types, groups differed in the number of sensory descriptions, χ^2^(2) = 7.21, *p* = 0.027, η^2 =^ 0.02. Specifically, the PTSD group reported fewer sensory details than trauma-exposed controls (*p* = 0.024, *r* = −0.17) but not healthy controls (*p* = 0.254, *r* = −0.11), whereas the two control groups did not differ from one another (*p* = 0.254). This was accentuated when examining the proportion of details, χ^2^(2) = 15.23, *p* < 0.001, η^2 =^ 0.04, such that the PTSD group reported a smaller proportion of sensory details than both healthy (*p* = 0.035, *r* = −0.17) and trauma-exposed controls (*p* < 0.001, *r* = −0.25), which did not differ (*p* = 0.083). No group differences were found for spatial references, χ^2^(2) = 1.90, *p* = 0.386, entities present, χ^2^(2) = 0.12, *p* = 0.943, or thoughts, emotions, and actions, χ^2^(2) = 5.83, *p* = 0.089.

**Table 2:** Scene construction task performance.

Variable	PTSD	Trauma-exposed controls	Healthy controls	Sig.
Overall experiential index	44.23 (12.05)	44.36 (8.27)	43.61 (8.83)	
Scored content (transcripts)				
Total details	22.89 (6.25)	23.23 (4.04)	23.18 (4.62)	
Spatial references	5.00 (2.23)	4.63 (2.11)	4.82 (2.11)	
Entities present	5.98 (1.77)	6.14 (1.41)	6.10 (1.55)	
Sensory descriptions	5.56 (1.99)	6.26 (1.50)	6.14 (1.44)	*
Thoughts, emotions, and actions	6.35 (1.70)	6.09 (1.70)	6.13 (1.51)	
Scorer rating	6.91 (2.33)	6.61 (1.69)	6.73 (2.02)	
Subjective ratings				
Presence	4.10 (1.15)	4.08 (0.89)	3.76 (1.07)	*
Salience	4.00 (1.02)	3.92 (0.99)	3.72 (0.99)	
Spatial coherence index	4.27 (2.74)	4.56 (2.79)	3.70 (2.95)	*

*p < 0.05.

For subjective ratings, group differences were found for presence, χ^2^(2) = 8.80, *p* = 0.013, η^2 =^ 0.02, and spatial coherence, χ^2^(2) = 6.92, *p* = 0.031, η^2 =^ 0.01, but not perceived salience, χ^2^(2) = 4.80, *p* = 0.091. Specifically, healthy controls reported lower presence than trauma-exposed controls (*p* = 0.030, *r* = 0.14) and those with PTSD (*p* = 0.029, r = 0.19), with no difference between trauma-exposed groups (*p* = 0.439). Trauma-exposed controls reported higher spatial coherence than healthy controls (*p* = 0.026, *r* = 0.15), however the PTSD group did not significantly differ from either trauma-exposed (*p* = 0.480) or healthy controls (*p* = 0.480).

### 3.3 Multiple linear regressions

Significant models are summarized in Table 3; see Supplementary Materials for full model summaries of all models. The model predicting overall scene construction performance through the Experiential Index was significant, *R^2^_adj_* = 0.28, *F* (7,31) = 3.15, *p* = 0.012, with cognitive ability being the only significant predictor (*β* = 0.47, *p* < 0.001). For specific detail types, the only significant model was for thoughts, emotions and actions, *R^2^_adj_* = 0.21, *F* (7,31) = 2.40, *p* = 0.044. Significant predictors were cognitive ability (β = 0.07, *p* = 0.006) and avoidance symptoms (β = 0.29, *p* = 0.049). The model predicting quality was also significant, *R^2^_adj_* = 0.27, *F* (7,31) = 2.98, *p* = 0.016, with cognitive ability (β = −0.11, *p* < 0.001) and depression symptoms (β = −0.08, *p* = 0.048) being significant predictors. Models for entities present, sensory descriptions, and spatial references were not significant, as were models for subjective ratings such as presence, salience, or spatial coherence.

**Table 3:** Summary of significant linear regression models.

Dependent variable	Predictor	*B*	*SE*	*t*	*p*
Experiential index	CPSS – arousal and reactivity	−0.03	0.45	−0.07	0.949
	CPSS – avoidance	0.76	0.76	1.01	0.321
	CPSS – cognition and mood	−0.51	0.41	−1.23	0.229
	CPSS – intrusions	0.16	0.54	0.29	0.776
	BDI-II	−0.27	0.17	−1.63	0.114
	Shipley – total score	0.47	0.12	3.84	<0.001
	BSI-53 – global severity index	4.30	3.14	1.37	0.180
Thoughts, emotions, and actions	CPSS – arousal and reactivity	−0.08	0.08	−0.91	0.371
	CPSS – avoidance	0.29	0.14	2.05	0.049
	CPSS – cognition and mood	−0.02	0.08	−0.27	0.787
	CPSS – intrusions	−0.04	0.10	−0.43	0.674
	BDI-II	−0.03	0.03	−0.80	0.429
	Shipley – total score	0.07	0.02	2.93	0.006
	BSI-53 – global severity index	0.46	0.59	0.79	0.437
Quality rating	CPSS – arousal and reactivity	0.03	0.10	0.33	0.746
	CPSS – avoidance	0.10	0.17	0.55	0.585
	CPSS – cognition and mood	−0.12	0.10	−1.17	0.250
	CPSS – intrusions	0.01	0.13	0.06	0.950
	BDI-II	−0.08	0.04	−2.06	0.048
	Shipley – total score	0.11	0.03	3.81	<0.001
	BSI-53 – global severity index	1.35	0.72	1.87	0.071

Each dependent variable represents its own linear regression analysis. See Supplementary Materials for reports of all models.

## 4 Discussion

Contrary to previous findings in adults (Marlatte et al., 2022), adolescents with PTSD did not display overall scene construction deficits and instead reported fewer sensory details in their imagined scenes compared to the control groups. Similarly, PTSD severity did not predict scene construction ability; instead, cognitive ability predicted several aspects of scene construction performance (i.e., Experiential Index, quality as rated by an external scorer, and details describing thoughts, emotions, and actions). Imagining more thoughts, emotions, and actions details was also associated with greater severity of avoidance symptoms, and imagining scenes of greater quality was related to reduced depression symptoms. Participants who had experienced a traumatic event reported feeling more present within their imagined scenes compared to healthy controls, and trauma-exposed controls also reported greater spatial coherence. Together, these results suggest that overall deficits in scene construction are not apparent in adolescence; however, changes in sensory imagery are. A consequence of such paucity in sensory imagery may be the observed increase in imagining person-related details. These results provide insights into changes in hippocampal-dependent functions across different populations with PTSD and can inform future interventions.

### 4.1 Adolescent PTSD is associated with deficits in sensory imagery

These findings indicate that deficits in imagining neutral scenes that are spatially coherent and detail rich, as seen in adult-onset PTSD, are not apparent during developmental PTSD. Instead, adolescent PTSD is associated with changes in how individuals experience neutral sensory imagery. Notably, the sensory imagery deficits were not associated with symptom severity in any specific PTSD symptom cluster, nor were they associated with depression severity, overall distress, or cognitive ability. Together, this suggests that sensory imagery deficits present in adolescent PTSD are associated with the broader clinical profile of the disorder rather than to specific core features or related, comorbid psychopathologies.

The few prior studies that have examined mental imagery in adolescent PTSD directly focused on affective content and noted differences in frequency and vividness of negative, as compared to positive, mental images (Steil et al., 2022). Of potential relevance to imagery are findings that trauma impacts somatic sensory processing (Kearney and Lanius, 2022). Childhood trauma has specifically been found to impact multisensory integration (Howard et al., 2020), defined as the ability to process, integrate, and organize input from our body and the environment to effectively interact with our surroundings (Ayres, 1972). Such processing is foundational for higher cognitive operations that rely on this input, such as memory, spatial navigation, social cognition, goal-oriented action, and even one’s sense of self (Harricharan et al., 2021). Similarly, impaired multisensory integration after childhood trauma has been associated with nervous system dysregulation (Howard et al., 2020), in alignment with embodied neuroscience theories of PTSD that emphasize how somatic sensory processing dysfunction has cascading influences on physiological arousal, affect, and higher-level cognition (Kearney and Lanius, 2022). Together, these results suggest that adolescents present a unique pattern of PTSD-related deficits in scene construction that exclusively involves impaired sensory processing when imagining neutral scenes, which may underpin subsequent trauma-related symptomatology.

### 4.2 Cognitive ability is critical in both scene construction ability and PTSD development

Cognitive ability was the strongest predictor of overall scene construction performance as measured by the Experiential Index. Our measure of cognitive ability is used to index different aspects of intelligence, reflecting the capacity for flexible reasoning and problem-solving. Previous work has shown detail generation in future thinking relies on a similar construct, cognitive flexibility (Addis et al., 2016), defined as the ability to dynamically adapt one’s thinking or behavior in response to changing environments. Future thinking also implicates frontal cognitive control regions more than episodic recollection (Benoit and Schacter, 2015), and greater cognitive flexibility during future thinking is associated with reduced hippocampal-dependent episodic reliving (Roberts et al., 2017). Together, these findings highlight the important role of cognitive ability and flexibility in both scene construction and future thinking, which may not be as hippocampally-dependent as memory recall.

Intelligence has previously been linked with PTSD development. A review of studies in adults (Buckley et al., 2000) identified lower intelligence as a potential vulnerability factor for later developing PTSD, and higher intelligence in childhood may act as a protective factor against PTSD development after later trauma exposure in adolescence (Breslau et al., 2006). In line with this, we have found that adults with PTSD showed both deficient scene construction performance and lower intelligence (Marlatte et al., 2022). Interestingly, here, adolescents with PTSD did not differ in intelligence from their controls, which may explain why no overall scene construction deficits were found. Notably, differences in fluid intelligence have not been seen previously in large-scale community (Keyes et al., 2017) or clinical samples (Saigh et al., 2006) of adolescents with PTSD. As flexible future problem solving is associated with greater hippocampal-vmPFC connectivity maturation into adulthood (Calabro et al., 2020), PTSD-related differences in intelligence—and therefore associated deficits in scene construction—may both emerge in adulthood instead.

### 4.3 Imagining person-related details may be a compensation mechanism

Those with PTSD imagined fewer sensory details, but also more details related to thoughts, emotions, and actions if they had greater avoidance symptoms. Imagining more of these person-related details may therefore be a compensation mechanism to avoid episodic reliving and the imagining of more detail-rich episodes, or it may simply reflect what is more accessible to imagine. Such a trade-off was similarly noted in adults with PTSD who imagined fewer spatial details (Marlatte et al., 2022). Whereas sensory processing develops in childhood, the development of spatial processing continues into early adulthood (Ruggiero et al., 2016), which may be why spatial deficits were not noted in adolescent PTSD here. Imagining person-related details also requires less relational processing which relies on the hippocampus (Wiebels et al., 2020), and therefore may be a common compensation strategy for those with PTSD across development. Future research should clarify whether reporting more thoughts, emotions, and actions in this task is either a narrative strategy for those high in avoidance, reflects what is accessible to imagine, or potentially both; such insights can inform the application of future interventions.

### 4.4 Adolescent PTSD is associated with differences in embodiment even within imagination

Participants who had experienced trauma reported feeling more present within their scenes; however, those with PTSD paradoxically also reported fewer sensory details. Together, this indicates that adolescents with PTSD may have a reduced embodied experience in their imagination. Their threshold of subjective presence may be lower than that of healthy controls, paradoxically resulting in higher subjective ratings of presence despite lower objective measurements of sensory experience. Episodic memory is still developing during adolescence (Mechie et al., 2021), including what subjectively constitutes as a present episode, which is presumably on a continuum. These individuals may therefore have fewer highly present experiences to compare to, given their trauma history, leading to a lower overall threshold for what is considered a highly present mental simulation. Further, experiencing traumatic experiences during adolescence, especially those that are interpersonal in nature as was common for our sample, is related to greater avoidance responses such as dissociation (Brand et al., 2012; van Dijke et al., 2015) even in adolescence (Putnam, 2009). Although not measured here, the greater likelihood of utilizing such avoidance strategies may further impact one’s ability to be embodied and present, both in-the-moment and in one’s imagination. Future work should qualitatively examine the narratives of episodic simulations to further elucidate if and how objective measurements of sensory processing and episodic reliving differ from subjective reports as provided through scale ratings, as well as how this relates to dissociative symptoms.

It is of note that trauma-exposed controls also expressed greater presence but without the sensory imagining deficits observed in those with PTSD. This may be a sign of resiliency and suggests that promoting both subjective and objective aspects of sensory experiencing, such as in somatic therapy or sensory modulation, may be an especially relevant treatment opportunity for adolescents who have experienced trauma. Further, this provides initial evidence for the relevance of applied interventions such as memory specificity training (MeST; Raes et al., 2009), which was developed based on the over-general memory effects noted in depression and post-traumatic stress disorder in adults. With the MeST, individuals are asked to focus on sensory, perceptual, and contextual details of episodic memories to improve the specificity of memory recall and in turn reduce psychopathological symptoms (Barry et al., 2021). Although limited research has been done on this intervention in adolescents (Pile et al., 2021), our findings suggest adolescents with PTSD may especially benefit from interventions like the MeST with a particular focus on the sensory aspects of episodes. Applying the MeST to future or hypothetical, rather than past, episodes may also be of particular benefit to this age-group. However, whether the focus should be on improving the ability to bring sensory details to mind or increasing one’s tolerance to sensory experience, even within one’s imagination, is an open question that should be explored.

### 4.5 When might hippocampal-dependent behavioral deficits be associated with PTSD?

Given that reduced hippocampal volume and deficits in hippocampal-dependent processing are robustly seen in adults with PTSD, this leads to the question of why such differences are not consistently present in adolescence. One theory is that decreases in hippocampal volume are due to the neurotoxic effects of stress, which may take time and repeated experiences to develop (Lupien et al., 2009; but see, e.g., Kremen et al., 2012). Such processes may interact with normal trajectories of hippocampal development, where there is an increase in hippocampal grey matter volume into mid-adolescence followed by selective loss through pruning into adulthood (Tamnes et al., 2018). Further, there are mixed findings regarding the relationship between hippocampal volumes and memory performance during typical development (Van Petten, 2004; but see Botdorf et al., 2022) suggesting a nonlinear relationship between hippocampal structure and cognitive performance across the lifespan. Indeed, episodic memory development has been found to be non-linear approaching adulthood (Mechie et al., 2021) which may be masking differences in mental simulation ability that become present later on. Adolescence and associated neural pruning may therefore be a critical period for structural and behavioral changes to emerge in PTSD, similar to other psychopathologies (Paus et al., 2008; Sakai, 2020).

To our knowledge, no study has examined scene construction or episodic future thinking in this population; however, previous studies of mental simulation have reported deficits in autobiographical memory in adolescents with both depression and trauma-exposure (Hitchcock et al., 2014). These studies differ in multiple ways: previous studies have typically used the autobiographical memory test, which categorizes memories broadly as episodic (or not) rather than more specific methods that quantify categories of narrative details, similar to the presently used task. Previous paradigms also prompt with valenced cues, whereas the current task used (presumably) neutral cues. Finally, most of the prompts within the scene construction task were apersonal, which may be easier for participants to imagine.

### 4.6 Limitations

Several additional measures would strengthen the conclusions of these behavioral findings. First, additional neuroimaging would allow assessment, rather than inference, of the relationship between scene construction differences with hippocampal volume. Additionally, a measurement of dissociation, such as the Multidimensional Inventory of Dissociation – Adolescent Version (MID-60-A), would allow direct assessment of the potential relationship of developmental trauma, dissociation, and sensory experience during mental simulations.

Given that trauma-related cognitive and hippocampal changes may be smaller in community samples (Calem et al., 2017; Scott et al., 2015), replicating these findings with a clinical sample is necessary to ensure scene construction differences are due to clinical and hippocampal, rather than sample, differences. This may explain the contradictory findings between our original study finding robust difference in scene construction performance in adults with PTSD (Marlatte et al., 2022) sampled from a clinic, and our current results in adolescents sampled from the community. Indeed, PTSD is a heterogeneous disorder. Although our sample size was determined to be sufficiently powered *a priori*, it may still be too small to fully capture the range of disorder phenotypes that exist, including their associated patterns of neurocognitive deficits and developmental trajectories. Phenotypical differences may, in fact, partially explain the behavioral differences between the current findings and our previous study in adults (Marlatte et al., 2022). Examining mental simulation performance in a larger sample of adolescents with PTSD would confirm whether scene construction deficits are solely in sensory imagery and clarify if such deficits are consistent across a broad range of PTSD profiles. In addition, examining mental simulation performance longitudinally in adolescents with PTSD would elucidate whether overall scene construction deficits after adolescent trauma develop in adulthood, and if this is similar across PTSD profiles.

## 5 Conclusion

In conclusion, we found that the ability to imagine rich multimodal scenes in adolescence was associated with their cognitive ability rather than PTSD severity, as had been previously observed in adults. These results suggest that hippocampal-dependent deficits in scene construction are not apparent in adolescence. However, changes in sensory processing and embodiment are noted, which may be a target for intervention. Whether or not spatial processing deficits develop later in adulthood, or is a feature of adult-acquired PTSD, remains to be investigated.

## Data Availability

The raw data supporting the conclusions of this article will be made available by the authors, without undue reservation.
