# Supplementary material for: Scene construction deficits in adolescent PTSD are in sensory, rather than spatial, imagery
Source: Front Psychol. 2025 Aug 6;16:1589756. doi: 10.3389/fpsyg.2025.1589756 (PMC12369414; doi:10.3389/fpsyg.2025.1589756)
Supplement: Supplementary file 1 [file Supplementary_file_1.docx]

**Supplementary Materials**

- 1. **Scenario prompts**

Imagine you are lying on a deserted white sandy beach in a beautiful tropical bay. Describe the experience and surroundings in as much detail as possible using all of your senses.

Imagine something you will be doing this weekend but just give me one event. Describe the experience and surroundings in as much detail as possible using all of your senses.

Imagine how you will be spending next New Years Eve. Describe the experience and surroundings in as much detail as possible using all of your senses.

Imagine you are standing in the busy main hall of a museum containing many impressive exhibits. Describe the experience and surroundings in as much detail as possible using all of your senses.

Imagine you are standing by a small stream somewhere deep in a forest. Describe the experience and surroundings in as much detail as possible using all of your senses.

Imagine the next time you will meet a friend. Describe the experience and surroundings in as much detail as possible using all of your senses.

Imagine you are standing on the deck of a ship that is just pulling out of port on the beginning of a voyage. Describe the experience and surroundings in as much detail as possible using all of your senses.

Imagine you are standing in the middle of a bustling street market. Describe the experience and surroundings in as much detail as possible using all of your senses.

Imagine you are sitting, having something to eat in a restaurant*. Describe the experience and surroundings in as much detail as possible using all of your senses.

*adapted for adolescent participants from original: *Imagine you are sitting, having something to drink at a pub.*

**1.2 Subjective ratings, completed by participant**

### Thinking about the scene you just imagined, rate your sense of presence on a scale from 1 to 5:

| Did not feel like I was there at all |  |  |  | Felt strongly like I was really there |
| --- | --- | --- | --- | --- |
| **1** | **2** | **3** | **4** | **5** |

### Thinking about the scene you just imagined, rate your sense of perceived salience on a scale from 1 to 5:

| I could not really see anything |  |  |  | Extremely salient |
| --- | --- | --- | --- | --- |
| **1** | **2** | **3** | **4** | **5** |

**1.3 Spatial coherence index, completed by participant**

Thinking about the scene you just imagined, here is a list of phrases. Please read each of them and select the ones you think best describe your imagined scene. Pick as many as you think are relevant to you.

1. It was quite fragmented
2. I saw the scene in color
3. It was similar to looking at a picture or seeing it on TV
4. I could see individual details, but it didn’t all fit together as a whole scene
5. I would find it easy to answer questions about the scene
6. It wasn’t so much a scene as a collection of images
7. I was able to use some senses other than vision e.g. sound, smell
8. I could see it as one whole scene in my mind’s eye
9. I was able to think of details associated with the general theme
10. I would find it easy to give further details of the surroundings in the scene
11. It wasn’t a scene you could step into; it wasn’t really joined-up
12. I would find it easy to substitute an aspect of the scene for something else

**1.4 Scoring sample**

Prompt: Imagine something you'll be doing this weekend but just give me one event. Describe the experience and surroundings in as much detail as possible using all of your senses.

**PTSD_20401**

TEA SD SD TEA

I will be sleeping. / The week was very long / and busy / and I had nothing else to do / so I was

TEA EP TEA TEA TEA

trying to kill time. / And my parents / woke me up / and I yelled at them, / and then I went back /

EP TEA TEA TEA

to bed / and then I missed dinner time. / And then when I woke up, / it was the middle of the

TANGENTIAL

night. / Yeah, that's all.

**Healthy_19929**

TEA EP TEA

Okay. This weekend I’m going to work / on an art project / that I've wanted to start for a while. /

SPA TEA EP TEA TEA SD

So I go downstairs and / I grab / some of the materials. / I look around / and I grab / a few

EP TEA SD TEA

pieces / of paper / and I make sure that they're different / colors because, / and I take / some

EP TEA TEA SPA SPA TEA EP

magazines too, / so that I can collage. / And then I go / up to / my room / and I sit at / my desk /

TEA REPEAT TEA TEA TEA

and, and I pull out / the materials and / place them there. / And I start cutting. / I start flipping

REPEAT TEA EP TEA TEA

through / the magazine / and cutting out / the images / that I like /. I like to, I, I pick out the ones /

EP TEA EP TEA

that have flowers in them / cause I want to make / sort of a bouquet out of it. / And when I finish

TEA REPEAT TEA SD

picking them out, / I take out my / piece of paper / and I begin gluing stuff on. / And the, the glue

TEA SD SPA

smells good, actually. / And I want to add / more color / in the background / because it looks

TEA EP TEA EP

bland to me. / So I use some pastels / and I rub it all over / and it gets in my fingernails / and I

TEA SPA TEA TEA

don't like how it feels. / So I go to the washroom / and I wash it out, / and then I go back / and I, I

TEA TEA TEA

finish it up and / I do the finishing touches. / And then I don't, I don't really like how it looks, / so

TEA SPA

I, I hide it / in my drawer. Yeah. /

**1.5 Quality rating by external scorer**

On a scale of 0-10, how well did you feel the description evoked a detailed picture of the experience in your own mind:

| No picture at all |  |  |  |  |  |  |  |  |  | Vivid, extremely rich picture |
| --- | --- | --- | --- | --- | --- | --- | --- | --- | --- | --- |
| **0** | **1** | **2** | **3** | **4** | **5** | **6** | **7** | **8** | **9** | **10** |

**1.6 Summary of Group Differences on CPSS-V Subscales**

|  | **PTSD** | **Trauma-exposed Controls** | **Healthy Controls** | **Sig.** |
| --- | --- | --- | --- | --- |
| Arousal and reactivity | 13.29 (4.96) | 3.16 (3.99) | 1.00 (2.22) | *** |
| Avoidance | 5.00 (1.29) | 1.42 (1.87) | 0.29 (0.73) | *** |
| Changes in cognition and mood | 17.29 (4.54) | 3.53 (3.61) | 0.64 (1.34) | *** |
| Intrusions | 12.14 (4.02) | 2.37 (2.17) | 0.43 (0.85) | *** |
| Impairment | 11.00 (6.48) | 1.89 (2.85) | 0.14 (0.53) | *** |
| Number of kinds of traumatic events | 3.00 (1.16) | 1.95 (1.31) | -- | *** |

**Note:** *** p < .001

**1.7 Group differences in demographics, psychopathology and cognitive ability**

Groups did not differ in terms of age, χ^2^(2) = 5.88, *p* = .053, η^2 =^ .11. or education, χ^2^(2) = 4.13, *p* = .127, η^2 =^ .06. A Fishers Exact test found no differences in the proportion of male and female participants across groups, *p* = .703.

Groups differed based on their total PTSD symptom severity, χ^2^(2) = 23.38, *p* < .001, η^2 =^ .58. Adolescents in the PTSD group had overall greater PTSD symptom severity than the trauma-exposed group (*p* = .003, *r* = .62) and healthy controls (*p* < .001, *r* = 1.00), who reported symptoms based on their most stressful experience which did not qualify as a Criterion A stressor. Trauma-exposed controls also had greater symptom severity than healthy controls (*p* = .019, *r* = .41).

Groups also differed on depression severity, χ^2^(2) = 7.95, *p* = .019, η^2 =^ .16. Adolescents in the PTSD group had greater depression symptom severity than trauma-exposed controls (*p* = .027, *r* = .54) and healthy controls (*p* = .021, *r* = .53). There were no differences between control groups (*p* = .892).

Groups did not differ on state anxiety, χ^2^(2) = 4.91, *p* = .086, but did differ on trait anxiety, χ^2^(2) = 9.55, *p* = .009, η^2 =^ .20. Adolescents in the PTSD group had higher trait anxiety than both trauma-exposed (*p* = .018, *r* = .51) and healthy controls (*p* = .008, *r* = 0.66), which did not differ (*p* = .494).

Groups were found to differ on overall distress associated with psychopathological symptoms, χ^2^(2) = 12.42, *p* = .002, η^2 =^ .28. Adolescents in the PTSD group had greater reported distress than trauma-exposed controls (*p* = .003, *r* = .63) and healthy controls (*p* = .002, *r* = 0.76), however there were no differences between control groups (*p* = .599).

For cognitive flexibility, no differences were found between groups on verbal fluency, χ^2^(2) = 0.96, *p* = .618, or abstraction, χ^2^(2) = 0.23, *p* = .893.

**1.8 Linear regression model summaries**

Experiential Index: *R^2^_adj_* = .284, *F*(7,31) = 3.15, *p* = .012

| **Predictor** | **β** | **SE** | ***t*** | ***p*** |
| --- | --- | --- | --- | --- |
| CPSS – arousal and reactivity | -0.03 | 0.45 | -0.07 | .949 |
| CPSS - avoidance | 0.76 | 0.76 | 1.01 | .321 |
| CPSS – changes in cognition and mood | -0.51 | 0.41 | -1.23 | .229 |
| CPSS – intrusions | 0.16 | 0.54 | 0.29 | .776 |
| BDI-II | -0.27 | 0.17 | -1.63 | .114 |
| Shipley – total score | 0.47 | 0.12 | 3.84 | < .001 |
| BSI-53 – global severity index | 4.30 | 3.14 | 1.37 | .180 |

Quality: *R^2^_adj_* = .27, *F*(7,31) = 2.98, *p* = .016

| **Predictor** | **β** | **SE** | ***t*** | ***p*** |
| --- | --- | --- | --- | --- |
| CPSS – arousal and reactivity | 0.03 | 0.10 | 0.33 | .746 |
| CPSS – avoidance | 0.10 | 0.17 | 0.55 | .585 |
| CPSS – changes in cognition and mood | -0.12 | 0.10 | -1.17 | .250 |
| CPSS – intrusions | 0.01 | 0.13 | 0.06 | .950 |
| BDI-II | -0.08 | 0.04 | -2.06 | .048 |
| Shipley – total score | 0.11 | 0.03 | 3.81 | < .001 |
| BSI-53 – global severity index | 1.35 | 0.72 | 1.87 | .071 |

Total Details: *R^2^_adj_* = .19, *F*(7,31) = 2.26, *p* = .056

| **Predictor** | **β** | **SE** | ***t*** | ***p*** |
| --- | --- | --- | --- | --- |
| CPSS – arousal and reactivity | 0.09 | 0.25 | 0.38 | .701 |
| CPSS – avoidance | 0.40 | 0.42 | 0.95 | .348 |
| CPSS – changes in cognition and mood | -0.35 | 0.23 | -1.52 | .138 |
| CPSS – intrusions | -0.01 | 0.30 | -0.03 | .979 |
| BDI-II | -0.10 | 0.09 | -1.07 | .294 |
| Shipley – total score | 0.22 | 0.07 | 3.18 | .003 |
| BSI-53 – global severity index | 2.28 | 1.74 | 1.31 | .200 |

Spatial references: *R^2^_adj_* = .17, *F*(7,31) = 2.01 *p* = .077

| **Predictor** | **β** | **SE** | ***t*** | ***p*** |
| --- | --- | --- | --- | --- |
| CPSS – arousal and reactivity | 0.10 | 0.10 | 1.01 | .320 |
| CPSS – avoidance | -0.03 | 0.17 | -0.19 | .849 |
| CPSS – changes in cognition and mood | -0.13 | 0.09 | -1.34 | .190 |
| CPSS – intrusions | 0.03 | 0.12 | 0.27 | .791 |
| BDI-II | -0.09 | 0.04 | -2.46 | .020 |
| Shipley – total score | 0.08 | 0.03 | 2.93 | .006 |
| BSI-53 – global severity index | 1.33 | 0.71 | 1.87 | .071 |

Thoughts, emotions, and actions: *R^2^_adj_* = .21, *F*(7,31) = 2.40, *p* = .044

| **Predictor** | **β** | **SE** | ***t*** | ***p*** |
| --- | --- | --- | --- | --- |
| CPSS – arousal and reactivity | -0.08 | 0.08 | -0.91 | .371 |
| CPSS – avoidance | 0.29 | 0.14 | 2.05 | .049 |
| CPSS – changes in cognition and mood | -0.02 | 0.08 | -0.27 | .787 |
| CPSS – intrusions | -0.04 | 0.10 | -0.43 | .674 |
| BDI-II | -0.03 | 0.03 | -0.80 | .429 |
| Shipley – total score | 0.07 | 0.02 | 2.93 | .006 |
| BSI-53 – global severity index | 0.46 | 0.59 | 0.79 | .437 |

Sensory descriptions: *R^2^_adj_* = .07, *F*(7,31) = 1.43, *p* = .229

| **Predictor** | **β** | **SE** | ***t*** | ***p*** |
| --- | --- | --- | --- | --- |
| CPSS – arousal and reactivity | 0.01 | 0.08 | 0.11 | .913 |
| CPSS – avoidance | 0.01 | 0.13 | 0.05 | .958 |
| CPSS – changes in cognition and mood | -0.10 | 0.07 | -1.46 | .156 |
| CPSS – intrusions | 0.03 | 0.09 | 0.30 | .764 |
| BDI-II | 0.03 | 0.03 | 0.81 | .424 |
| Shipley – total score | 0.03 | 0.02 | 1.29 | .206 |
| BSI-53 – global severity index | 0.29 | 0.53 | 0.54 | .594 |

Entities present: *R^2^_adj_* = .06, *F*(7,31) = 1.32, *p* = .275

| **Predictor** | **β** | **SE** | ***t*** | ***p*** |
| --- | --- | --- | --- | --- |
| CPSS – arousal and reactivity | 0.06 | 0.07 | 0.87 | .392 |
| CPSS – avoidance | 0.13 | 0.12 | 1.17 | .253 |
| CPSS – changes in cognition and mood | -0.10 | 0.06 | -1.60 | .121 |
| CPSS – intrusions | -0.03 | 0.08 | -0.31 | .760 |
| BDI-II | 0.00 | 0.03 | -0.14 | .891 |
| Shipley – total score | 0.04 | 0.02 | 2.17 | .038 |
| BSI-53 – global severity index | 0.20 | 0.48 | 0.42 | .680 |

Presence: *R^2^_adj_* = -.14, *F*(7,31) = 0.35, *p* = .925

| **Predictor** | **β** | **SE** | ***t*** | ***p*** |
| --- | --- | --- | --- | --- |
| CPSS – arousal and reactivity | -0.02 | 0.05 | -0.36 | .720 |
| CPSS – avoidance | 0.05 | 0.09 | 0.55 | .588 |
| CPSS – changes in cognition and mood | 0.01 | 0.05 | -0.10 | .924 |
| CPSS – intrusions | 0.03 | 0.06 | 0.53 | .559 |
| BDI-II | -0.01 | 0.02 | -0.60 | .555 |
| Shipley – total score | 0.01 | 0.01 | 0.39 | .700 |
| BSI-53 – global severity index | -0.05 | 0.36 | -0.13 | .900 |

Salience: *R^2^_adj_* < .001, *F*(7,31) = 1.00, *p* = .449

| **Predictor** | **β** | **SE** | ***t*** | ***p*** |
| --- | --- | --- | --- | --- |
| CPSS – arousal and reactivity | -0.01 | 0.05 | -0.28 | .780 |
| CPSS – avoidance | -0.01 | 0.08 | -0.12 | .905 |
| CPSS – changes in cognition and mood | 0.01 | 0.04 | 0.31 | .759 |
| CPSS – intrusions | 0.05 | 0.05 | 0.95 | .350 |
| BDI-II | -0.02 | 0.02 | -1.00 | .328 |
| Shipley – total score | 0.02 | 0.01 | 1.55 | .131 |
| BSI-53 – global severity index | -0.08 | 0.31 | -0.25 | .807 |

Spatial Coherence: *R^2^_adj_* = -.04, *F*(7,31) = 0.78, *p* = .612

| **Predictor** | **β** | **SE** | ***t*** | ***p*** |
| --- | --- | --- | --- | --- |
| CPSS – arousal and reactivity | -0.16 | 0.16 | -1.00 | .322 |
| CPSS – avoidance | 0.26 | 0.27 | 0.98 | .336 |
| CPSS – changes in cognition and mood | 0.04 | 0.14 | 0.25 | .807 |
| CPSS – intrusions | 0.02 | 0.19 | 0.12 | .909 |
| BDI-II | -0.03 | 0.06 | -0.54 | .596 |
| Shipley – total score | 0.05 | 0.04 | 1.17 | .252 |
| BSI-53 – global severity index | 0.12 | 1.10 | 0.11 | .916 |
